# Behavioral Effects of Stimulated Dopamine Release and D2-like Receptor Displacement in Parkinson’s Patients with Impulse-Control Disorder

**DOI:** 10.3390/ijms26083866

**Published:** 2025-04-19

**Authors:** Megan A. Aumann, Sean J. Lee, Alexander K. Song, Kaitlyn R. O’Rourke, Paula Trujillo, Yan Yan, Hakmook Kang, Daniel O. Claassen

**Affiliations:** 1 Department of Neurology, Division of Behavioral and Cognitive Neurology, Vanderbilt University Medical Center, Nashville, TN 37232, USA; megan.aumann.1@vumc.org (M.A.A.); sean.j.lee.1@vumc.org (S.J.L.); alexander.k.song.1@vumc.org (A.K.S.); katie.orourke@vumc.org (K.R.O.); paula.trujillo@vumc.org (P.T.); 2 Department of Biostatistics, Vanderbilt University Medical Center, Nashville, TN 37232, USA; yan.yan.1@vanderbilt.edu (Y.Y.); h.kang@vumc.org (H.K.)

**Keywords:** dopamine, amphetamine, fallypride, Parkinson’s, impulsivity, mood

## Abstract

Dysregulated dopamine (DA) release in the mesocorticolimbic circuit is noted in Parkinson’s disease (PD) patients with impulsive and compulsive behaviors (ICBs). However, the effect of acute DA release on mood, the localization of this process, and the phenotypic differences in patients with ICB remain unknown. We applied a placebo-controlled dextro-amphetamine (dAMPH) study in 20 PD patients: 10 with ICBs (PD-ICB) and 10 without (PD-C). Subjective mood experiences were measured with well-described self-reported measures including the Positive and Negative Affect Scale (PANAS), Drug Effects Questionnaire (DEQ), and Amphetamine Interview Rating Scale (AIRS). D2-like receptor availability was measured as non-displaceable binding potential (BP_ND_) using PET imaging with the high-affinity D2/3 receptor ligand [^18^F]-fallypride. Among all the subjects, dAMPH increased the PANAS positive, DEQ feel, DEQ high, and AIRS total scores. Increases in the PANAS positive and AIRS total scores were greater in the PD-ICB cohort. A mixed-effects model correlated these questionnaire changes with dAMPH-induced reductions in BP_ND_ in the ventral striatum (VS), caudate, amygdala, and caudo-medial orbitofrontal cortex. The baseline caudate, VS, and amygdala BP_ND_ positively correlated with lower on-dAMPH PANAS positive scores. Elevated mood symptoms of acute dAMPH administration in PD are linked to DA release in the mesocorticolimbic regions. Distinctions in behavioral effects among PD-ICB subjects emphasize that dysregulated striatal and extra-striatal DA-ergic networks alter mood responses to stimulated DA release and may also contribute to behavioral changes resulting from DA-targeting therapies in PD.

## 1. Introduction

Patients with Parkinson’s disease (PD) experience a variety of non-motor symptoms that include psychiatric and behavioral changes, among which apathy, anxiety, and depression are the most common [1,2]. While necessary for symptomatic control of motor dysfunction, treatments that target dopamine (DA) can also modify behavioral affect. D2 and D3 receptor agonists have evidence of improving depressive symptoms [3]. However, DA agonist (DAA) usage is the strongest risk factor for the development of impulsive and compulsive behaviors (ICBs), which arise in about one-third of treated PD patients [4,5,6,7]. Defined as pathologic failures to resist urges to perform acts regardless of their negative consequences [8,9], ICBs have been linked to altered ventral striatal D2-like receptor (D2-R) expression and dysregulated mesocorticolimbic DA release, emphasizing the influence of the DA-ergic system in regulating mood in PD [10,11,12,13].

The dorsal and ventral DA networks are differentially impacted in PD. Motor symptoms such as tremor and bradykinesia clearly respond to DAA therapies, which modulate dorsally located structures such as the substantia nigra and dorsal striatum, which are more likely to be impacted early in the disease course. The relative preservation of ventral DA networks, especially early in the course of PD, may predispose PD patients to ICBs as a result of DAA increasing DA neurotransmission in the ventral striatum (VS), putamen, and caudate head [10,14,15,16,17,18]. DA dysregulation contributions to ICBs may also occur extra-striatally, particularly in areas with known striatal connections such as the amygdala, caudo-medial orbitofrontal cortex (cmOFC), insula, and anterior cingulate cortex [10,19,20,21,22,23].

Pharmacologic challenge studies can provide important insights linking neurotransmitter changes to neuroanatomical circuits and behavioral responses. Amphetamine is commonly used to study DA neurotransmission through its combined ability to increase pre-synaptic DA release from stored vesicles and impair DA reuptake by inhibiting DA transporters (DAT) and promoting the DAT-mediated reverse transport of DA into the synaptic cleft [24]. Many behavioral studies have utilized dextro-amphetamine (dAMPH) to understand how acute DA release influences mood, affect, and physical sensations. Indeed, amphetamine has been consistently associated with increased feelings of vigor, elation, friendliness, and overall positive mood enhancement [25,26]. Although these mood effects have been associated with increased levels of DA [27], few studies have focused on where these effects localize, with one report of males but not females showing positive mood-associated DA release in the left substantia nigra [28], while another study did not show significant brain regions associated with the amphetamine-induced positive affect [29].

In this study, we performed a single-blinded, placebo-controlled dAMPH intervention, with concomitant D2-like receptor imaging, in a cohort of PD patients with and without ICBs. Our goals were to (1) assess dAMPH-mediated effects on mood in PD, (2) localize dAMPH-induced DA release, and (3) determine the relationship between baseline D2-R availability and dAMPH-induced effects on mood. The D2-R was quantified using positron emission tomography (PET) with [^18^F]-fallypride [29], a D2-like receptor-specific ligand that provides both striatal and extra-striatal assessments of the D2-like receptor’s non-displaceable binding potential (BP_ND_). Acute effects on mood were assessed using the Positive and Negative Affect Schedule (PANAS) [30,31,32], which assesses emotional affect in a two-dimensional model of mood; the Drug Effects Questionnaire (DEQ) [33,34], which assesses the acute subjective effects of addictive substances; and the Amphetamine Interview Rating Scale (AIRS) [35,36], which assesses the effects of amphetamine on mood and physical sensations. By examining the relationship between D2-R availability and subjective mood ratings in PD patients with and without ICB, our study provides insight into the neuroanatomical substrates of DA-ergic regulation of mood in PD.

## 2. Results

### 2.1. dAMPH Effects on Mood

**Figure 1:**
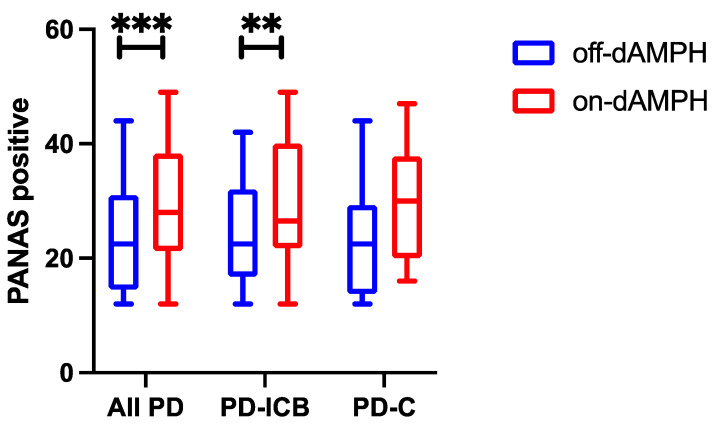
Box-and-whisker plots showing the median and quartile distribution for PANAS positive scores in all PD subjects (**left**) and split into PD-ICB (**middle**) and PD-C cohorts (**right**) in both off-dAMPH (blue) and on-dAMPH (red) conditions. ** Indicates statistically significant results after multiple comparisons correction at *p* < 0.05, and *** at *p* < 0.01.

We evaluated the effect of dAMPH on behavioral outcomes as measured by three complimentary scales that assess stimulant effects on mood. The scores reported in the placebo state are denoted as “off-dAMPH”, and those reported in the dAMPH state are denoted as “on-dAPMH”. dAMPH administration increased PANAS positive scores across all the PD subjects (Cohen’s d = 0.612; p_CORR_ = 0.002; Figure 1). When separated by ICB status, we found that this relationship was driven by the PD-ICB group, which showed greater changes when assessing the ON–OFF state (d = 0.660; p_CORR_ = 0.025) than in the PD-C group, whose ON–OFF differences were noticeable but did not survive multiple comparisons correction (d = 0.533; *p* = 0.026; p_CORR_ = 0.338; Figure 1). QUIP-RS ratings did not correlate with the PANAS positive response. No effect of dAMPH was observed for the PANAS negative subscale.

**Figure 2:**
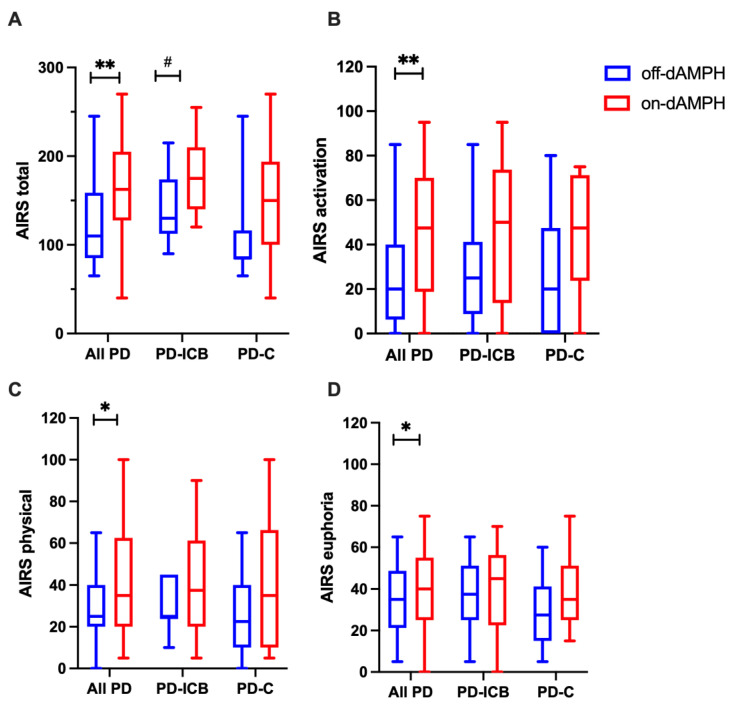
Box-and-whisker plots showing the median and quartile distribution for all PD subjects (**left**) and split into PD-ICB (**middle**) and PD-C cohorts (**right**) in both off-dAMPH (blue) and on-dAMPH (red) conditions for AIRS total (**A**), AIRS activation (**B**), AIRS physical (**C**), and AIRS euphoria (**D**) scores. * Indicates statistically significant results after multiple comparisons correction at *p* < 0.1 and ** at *p* < 0.05. # Indicates results significant at *p* < 0.05 before multiple comparisons correction.

When considering dAMPH-related changes in AIRS, the total scores increased significantly from placebo across all the participants (d = 0.829; p_CORR_ = 0.012) (Figure 2A). In a similar pattern to the PANAS positive scores, we observed more significant changes in the PD-ICB cohort (d = 0.623; *p* = 0.041) than in the PD-C cohort (d = 0.669; *p* = 0.066) (Figure 2A). Within AIRS, across all the PD subjects, dAMPH significantly increased scores in the activation (d = 0.645; p_CORR_ = 0.029), physical (d = 0.561; p_CORR_ = 0.061), and euphoria (d = 0.329; p_CORR_ = 0.088) subscales (Figure 2B, Figure 2C, Figure 2D, respectively). For these subscales, it did not appear that either the PD-ICB or the PD-C cohort responded differently to dAMPH (Figure 2B–D). Interestingly, while AIRS sleepiness scores did not change across all the participants (d = −2.75; p_CORR_ = 0.39), the PD-ICB cohort showed significant reductions in AIRS sleepiness (d = −0.628; p_CORR_ = 0.025), which was not seen in the PD-C group. No effect of dAMPH was observed for the AIRS depression or dysphoria subscales.

**Figure 3:**
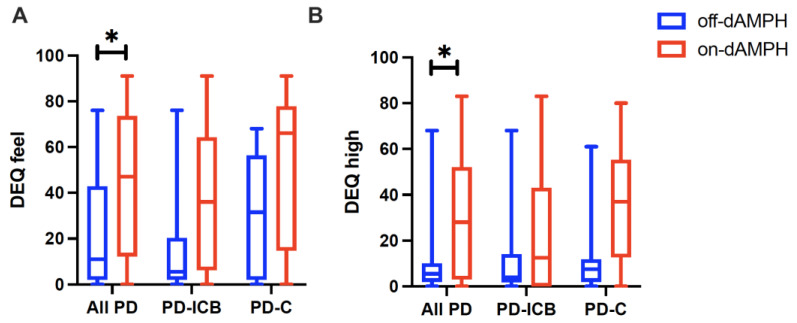
Box-and-whisker plots showing the median and quartile distribution for all PD subjects (**left**) and split into PD-ICB (**middle**) and PD-C cohorts (**right**) in both off-dAMPH (blue) and on-dAMPH (red) conditions for DEQ feel (**A**) and DEQ high (**B**) scores. * Indicates statistically significant results after multiple comparisons correction at *p* < 0.1. Spearman correlation indicating the relationship between change in AIRS depression scores, defined as (off-dAMPH–on-dAMPH)/off-dAMPH, related to QUIP-RS across all PD subjects.

Finally, when assessing dAMPH-induced changes in the DEQ subscales, we noted significant increases across all the PD subjects in DEQ feel (d = 0.793; p_CORR_ = 0.063) (Figure 3A) and DEQ high scores (d = 0.777; p_CORR_ = 0.086) (Figure 3B). However, neither the PD-ICB nor the PD-C cohort responded differently to dAMPH (Figure 3A and Figure 3B, respectively) in either subscale. QUIP-RS ratings did not correlate with the DEQ feel or high response. No effect of dAMPH was observed for the DEQ dislike, like, or want subscales.

### 2.2. Localization of DA Release Associations with Subjective Experiences

The effect of dAMPH on the non-displaceable binding potential (BP_ND_) and behavioral responses was assessed using a linear mixed-effects model; for quantification of dAMPH-induced displacement, see Table 2 in [10]. This analysis focused on the questionnaire subscales in which significant dAMPH-induced effects were noted among all the participants. Subscale score relationships with regional BP_ND_ changes can all be found in Table 1. Total AIRS scores significantly correlated with dAMPH-induced reductions in VS (ventral striatum) BP_ND_ (r = −0.011; p_CORR_ = 0.027)_,_ amygdala BP_ND_ (r = −0.001; p_CORR_ = 0.080), and cmOFC (caudo-medial orbitofrontal cortex) BP_ND_ (r = −0.006; p_CORR_ = 0.070). Among the subscales assessed, a significant correlation between AIRS activation scores and VS BP_ND_ was noted (r = −0.022; p_CORR_ = 0.059).

Although the following relationships did not survive multiple comparisons correction, we find these results noteworthy as similar ROIs appeared in multiple statistical analyses. The PANAS positive scores correlated with dAMPH-induced BP_ND_ reductions in the VS (r = −0.055; *p* = 0.034). The DEQ feel scores correlated with dAMPH-induced BP_ND_ reductions in the caudate head (r = −0.009; *p* = 0.020) and VS (r = −0.012; *p* = 0.031). The DEQ high scores correlated with dAMPH-induced BP_ND_ reductions in the caudate head (r = −0.008; *p* = 0.050). The AIRS activation scores correlated with dAMPH-induced BP_ND_ reductions in the caudate (r = −0.011; *p* = 0.046) and hypothalamus (r = −0.003; *p* = 0.041). The AIRS physical subscale scores correlated with BP_ND_ reductions in the caudate head (r = −0.014; *p* = 0.039), VS (r = −0.022; *p* = 0.019), cmOFC (r = −0.011; *p* = 0.050), and insula (r = −0.004; *p* = 0.036).

There were no significant correlations between any of the questionnaire scores and the BP_ND_ in the putamen, GP, SN, or ACC.

**Figure 4:**
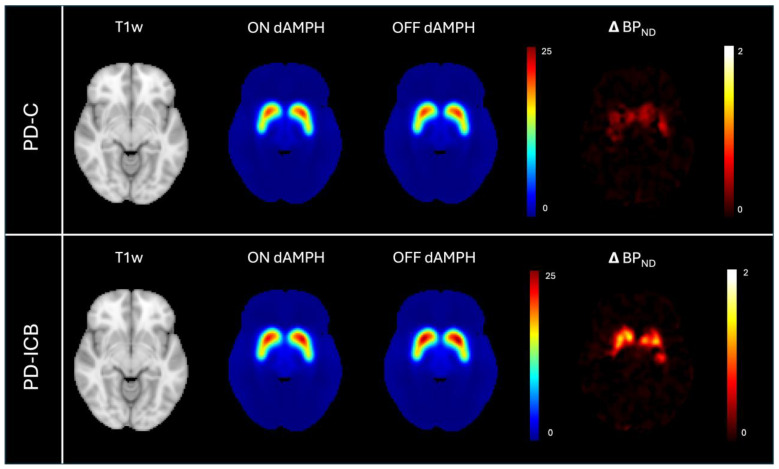
T1-weighted (T1w) magnetic resonance image (MRI) for anatomical reference associated with PET BP_ND_ maps in the amphetamine (ON dAMPH) and baseline (OFF dAMPH) conditions for both Parkinson’s patients without impulsive–compulsive behaviors (PD-C; **top row**) and with impulsive–compulsive behaviors (PD-ICB; **bottom row**). The average change in BP_ND_ for each group is shown on the far right as Δ BP_ND_ (OFF-ON dAMPH).

To appreciate where these behavioral changes localize in the brain, Figure 4 shows the average effect of dAMPH administration for each cohort, with binding potential (BP_ND_) maps following dAMPH administration (ON) and in the baseline (OFF) conditions, and the average difference between conditions expressed in a heat map.

### 2.3. Baseline D2-R Availability as a Predictor of Amphetamine Effects

**Figure 5:**
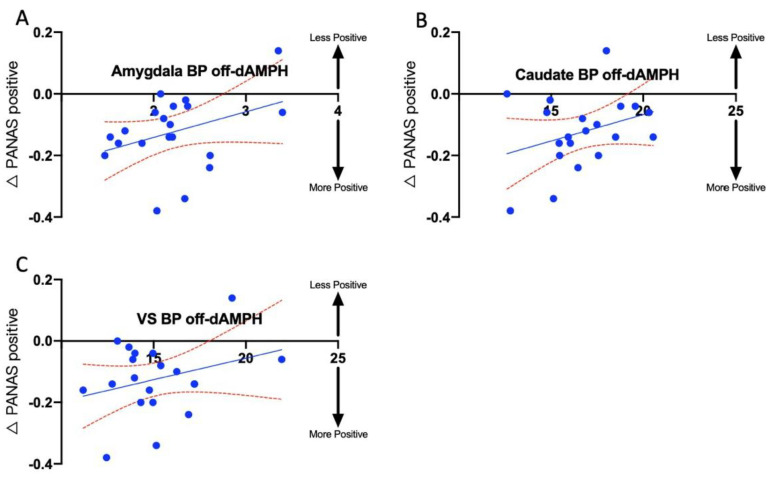
Spearman correlation indicating the relationship between change in PANAS positive scores, defined as (off-dAMPH-on-dAMPH)/off-dAMPH, related to baseline binding potential (BP) across all PD subjects for the amygdala (**A**), caudate (**B**), and ventral striatum (VS) (**C**). Blue dots indicate individual subjects; solid blue line indicates regression line; red dotted lines indicate 95% confidence interval.

Finally, we assessed the baseline (off-dAMPH) D2-R availability as a predictor of dAMPH-induced changes in subjective mood. We found significant positive associations between the changes in PANAS positive scores and the baseline BP_ND_ in the amygdala (R^2^ = 0.36; p_CORR_ = 0.091; Figure 5A), caudate head (R^2^ = 0.33; p_CORR_ = 0.091; Figure 5B), and VS (R^2^ = 0.37; p_CORR_ = 0.091; Figure 5C). This relationship indicates that a higher baseline BP_ND_ corresponded to a greater reduction in PANAS positive following dAMPH administration. We did not find associations involving DEQ feel, DEQ high, total AIRS, or any AIRS subscale scores and baseline BP_ND_ that survived multiple comparisons correction for this analysis. In addition, no significant associations involving any ROI other than the amygdala, caudate head, and VS were noted.

## 3. Discussion

In patients with PD, acute dAMPH administration induced positive mood effects localizing to the mesocorticolimbic structures, most significantly in the ventral striatum (VS), but also in the caudate head and the extra-striatal regions of the amygdala and cmOFC. While previous studies have implicated the VS and caudate in maintaining positive mood and affect in PD [37], we are the first to report contributions from the amygdala and cmOFC in this capacity. In PD patients, impulsivity correlated positively with dAMPH-induced depression, suggesting that dysregulated DA neurotransmission in the mesocorticolimbic structures may result in abnormal mood and behavioral symptoms. Finally, baseline D2-R availability in the mesocorticolimbic structures of the VS, caudate head, and amygdala inversely correlated with dAMPH-induced changes in positive mood responses; we interpret greater D2-R availability as more preserved DA-ergic networks.

To date, short-term dAMPH effects have been investigated only in healthy subjects, with affective changes including euphoria, feelings of drug effect, and positive mood [38,39,40]. Of note, [41] reports that, among healthy adults, dAMPH induced significant changes in ‘positive’ mood domains (e.g., ‘arousal’ and ‘drug high’) but had no net effect on ‘negative’ domains, a finding replicated here in a cohort of PD patients. Furthermore, a [^18^F]-fallypride study in healthy adults related dAMPH-induced DA receptor displacement in VS to attention and cognitive processing but not to affect [29]. This study extends previous [^18^F]-fallypride-based investigations of dAMPH effects by involving patients with known neuroanatomical defects (e.g., PD) and linking dAMPH-induced mood and behavioral effects to changes in D2-R availability in both striatal and extra-striatal regions. Here, we discuss these findings and the implications for future therapeutic interventions in PD.

### 3.1. Localization of dAMPH Effects

The focus on the effects of acute dAMPH administration in a PD population allows us to investigate DA release in the mesocorticolimbic network, as dorsal DA networks are essentially lesioned in PD. Since early motor manifestations of PD involve the progressive loss of dorsally located DA-ergic neurons, most notably in the midbrain and dorsal striatum, we did not expect significant DA release to occur in this network. This was corroborated by our mixed-effects model, which showed positive mood effects localizing chiefly to the VS, with similar trends in the caudate, amygdala, and cmOFC. Our results reinforce the findings from preclinical studies indicating a role for the mesocortical structures in affective and behavioral regulation [42,43]. Moreover, given the VS and caudate’s known roles in reward and habit formation [44,45,46], we accurately hypothesized that the acute mood effects of an addictive substance like amphetamine would correlate with DA release in the VS and caudate.

Importantly, our study found extra-striatal contributions to positive mood regulation among PD patients. The OFC sends dense projections to the amygdala, and both share bidirectional inputs to the hypothalamus. These three structures play complementary roles: the amygdala encodes information about emotional value; the hypothalamus coordinates peripheral emotional responses; and the OFC helps adapt behavior in relation to emotional cues [47,48]. These structures are likely involved in broad emotional valence rather than in one specific mood state, consistent with recent findings indicating that the amygdala and OFC are sensitive to positive emotion intensity [49]. Finally, while previous studies [23,50] have examined the role of the amygdala and the cmOFC, respectively, in healthy subjects’ responses to dAMPH, we are the first to explore how these structures contribute to mood regulation in PD. Overall, our results are consistent with a relatively intact mesocorticolimbic circuit in the early-to-mid stages of PD.

Interestingly, the PANAS and DEQ dAMPH-related score changes were associated with DA release from the caudate head and VS, whereas the AIRS changes correlated both in the striatum (caudate and VS) and extra-striatally in the amygdala and cmOFC. These differences could be explained by the content of the AIRS assessment, which interrogates more physical symptoms (e.g., alertness, dizziness) than the PANAS or the DEQ. The results show that the AIRS subscale scores were the highest in the activation and physical responses, indicating that, under the influence of dAMPH, PD patients feel not merely positive mood effects but increased levels of arousal. Both the amygdala and the OFC have been shown to play a role in arousal, and primate data support the importance of the OFC in modulating arousal in relation to emotional and social cues [51].

Together, our data suggest a striatal-fronto-cortical network of mood regulation in the presence of dAMPH, and the mesocorticolimbic circuit as a viable target for mood symptoms in PD. Moreover, findings in the amygdala and cmOFC emphasize the use of [^18^F]-fallypride as an assay for D2-R availability both within and outside the striatum and underscore how affective regulation occurs extra-striatally.

### 3.2. Impulsivity and Mood in PD

ICBs among PD patients are thought to emerge from DAA-induced increases in phasic DA release in structures such as the VS [10]. In otherwise healthy subjects, other studies have linked impulsivity to increased tonic levels of synaptic DA in the striatum [14,52,53]. Among ICB patients, an altered neurobiology of striatal DA networks may predispose patients to unwanted non-motor side effects from typical DA-modifying therapies. Predictably, we observed differing mood responses to dAMPH based on ICB status. Specifically, ICB+ patients showed greater dAMPH-induced elevations in positive mood than their ICB− counterparts as well as greater increases in AIRS total scores, indicating an overall elevation in multiple symptom domains, including physical and affective changes. Our data imply that increased tonic synaptic DA in the striatum may augment the pleiotropic effects of phasic DA release.

The net positive effect of dAMPH on mood remained consistent in both the PD-ICB and the PD-C cohorts. However, when taken as a continuous variable (e.g., QUIP-RS score), impulsivity was found to positively correlate with greater feelings of depression following acute dAMPH administration. Notably, we are not the first to observe a relationship between impulsivity and depression in PD; Scott et al. (2020) [54] observed that depression was most common alongside both apathy and ICB and cited a lack of motivational control as a potential unifier between negative mood and dysregulated behavior [55]. Our study suggests that, although acute dAMPH administration is related to an overall positive mood across the entire cohort, patients with sufficiently severe ICB may experience the opposite effect. These results echo prior reports of an inverted U-shaped relationship between baseline DA-R availability and mood responses to DAA therapy [56]. Mechanistically, since ICBs are associated with stronger striatal and extra-striatal phasic DA release [10], increased DA neurotransmission in the mesocortical areas involved in motivation and reward could paradoxically result in more depressive feelings—consistent with the ‘dopamine overdose hypothesis’ of mood. Finally, the fact that PD patients exhibit higher levels of impulsivity than healthy controls [55] highlights the importance of considering the baseline D2-R availability in the general PD population and not merely in those patients with diagnosed ICBs.

### 3.3. Baseline BP_ND_ as Predictors of Mood Effects

The results show an interesting relationship between BP_ND_ in the off-drug state and the PANAS positive subscale. All the subjects experienced greater dAMPH-induced increases in positive mood, with decreased baseline D2-R availability in the VS, caudate head, and amygdala. Our results could be explained by D2-R downregulation and neuronal death, both pathologic processes that worsen as PD progresses [57]; fewer receptors would increase the competition for binding spots, resulting in a lower BP_ND_. Additionally, because these processes tend to progress in a caudo-rostral fashion, it is likely that DA receptors in more rostral parts of the brain such as the VS are more preserved. Notably, Stark et al. (2018) found that, compared with age- and sex-matched controls, PD patients exhibit significantly lower BP_ND_ in the caudate and amygdala but not in the VS [58]. It is noteworthy that when assessing positive mood effects in relation to baseline D2-R availability, the structures of the VS, caudate, and amygdala were again implicated to subserve mood effects of dAMPH—mirroring our mixed-effects model. These results further underscore the role of DA in mood regulation and provide support for the mesocorticolimbic circuit in modulating affective responses to DA-ergic changes.

Our study had several limitations, most notably a sample size of 20. However, such a cohort size is not uncommon for PET studies, given the rigorous nature of these investigations. To mitigate, we employed a repeated-measures design and consistently used the covariates of age, sex, and UPDRS-III score in all the statistical analyses, with age and UPDRS-III scores combined into a principal component to reduce the number of covariates. Notably, the ROIs of the VS, caudate, and amygdala were found to significantly correlate with mood effects in two different statistical analyses, underscoring the consistency of our results. Another limitation was that the self-reported questionnaires relied on the patients’ insight into changes in mood and behavior; however, the responses were obtained in both off- and on-drug conditions so that each subject had a baseline score to serve as an internal control. Finally, D2-R availability is heterogenous in this population and relates to other factors besides mood such as patient age, disease severity, and disease duration [57]. However, we accounted for these factors in our statistical analyses, and our mixed-effects model used both off- and on-drug BP_ND_, accounting for each subject’s D2-R availability at baseline.

## 4. Materials and Methods

### 4.1. Population

The participants were recruited from the Vanderbilt University Medical Center Department of Neurology, and all completed written informed consent approved by the Vanderbilt University Institutional Review Board. The exclusion criteria included DAA therapy for >8 years, patient age <45 or >80 years, concomitant use of GABA-altering medications, comorbid neurological disease (e.g., stroke, dementia), diagnosis of an untreated mood disorder from the Diagnostic and Statistical Manual of Mental Disorders (5th ed.) [59], prior history of deep-brain stimulation surgical implant, and any other condition precluding MRI imaging.

In total, 20 participants diagnosed with idiopathic PD completed this study. Based on the diagnostic interview, 10 met the criteria for ICB disorder (PD-ICB), and 10 did not (PD-C). All the participants completed the Montreal Cognitive Assessment (MoCA) to assess global cognitive functioning (average score = 26.0), the Movement Disorders Society—Unified Parkinson’s Disease Rating Scale (MDS-UPDRS) [60,61,62] parts II and III to assess symptom severity, and the Questionnaire for Impulsive–Compulsive Disorders in Parkinson’s Disease Rating Scale (QUIP-RS) to quantify impulsive behaviors [63,64]. The groups were evenly matched for sex, age, disease duration, UPDRS-III score, and levodopa equivalent daily dose (Table 2). The PD-ICB cohort had significantly higher QUIP-RS (*p* = 0.038) scores than the PD-C cohort (Table 2).

### 4.2. Trait Impulsivity, Subjective Measures

Acute effects on mood were measured with well-described self-reported measures using the Positive and Negative Affect Schedule (PANAS) [30], which assesses emotional affect in a two-dimensional model of mood; the Drug Effects Questionnaire (DEQ) [33,34], which assesses the acute subjective effects of addictive substances; and the Amphetamine Interview Rating Scale (AIRS) [35], which assesses the effects of amphetamine on mood and physical sensations.

Subscale scores were calculated, including the 2 sub-domains of PANAS, 5 sub-domains of DEQ, and 6 sub-domains of AIRS. A percent change in the scores was defined as (score off-dAMPH score on-dAMPH)/maximum subscale score, which accounted for baseline scale scores rated as zero. Scores in PANAS positive and PANAS negative both ranged from 10 to 50. Scores in DEQ feel, DEQ high, DEQ dislike, DEQ like, and DEQ want ranged from 0 to 100 each. Scores in AIRS activation and AIRS depression both ranged from 0 to 120, AIRS physical from 0 to 180, AIRS euphoria from 0 to 80, AIRS dysphoria from 0 to 140, and AIRS sleepiness from 0 to 40.

### 4.3. MRI Acquisition

Magnetic resonance imaging (MRI) scans were acquired to provide high-resolution structural delineation for the quantification of [^18^F]-fallypride non-displaceable binding potential (BP_ND_). All the scans were completed with a 3.0 T Philips scanner using body coil transmission and 32-channel SENSE array reception. Structural images were acquired using a T_1_-weighted high-resolution anatomical scan (MPRAGE; spatial resolution = 1 × 1 × 1 mm^3^; TR/TE = 8.9/4.6 ms). MRI scans were obtained prior to PET scans on each patient’s first visit day.

### 4.4. PET Imaging, Data Processing

[^18^F]-fallypride was produced by the Vanderbilt Radiochemistry Core laboratory using the synthesis and quality-control procedures described in U.S. Food and Drug Administration IND 12,035. The PET scans were completed on a Philips Vereos PET-CT scanner with a 3D emission acquisition and a transmission attenuation correction. The images had an axial resolution of 4 mm and an in-plane resolution of 4.0 mm with a 5.8 mm FWHM. Following a bolus injection of 5.0 mCi [^18^F]-fallypride, serial scans were obtained for approximately 3.5 h. The subjects received two scans: one in the on-dAMPH state and another in the off-dAMPH state. PET image corrections and registration were performed as previously described [10,65]. The [^18^F]-fallypride BP_ND_ was quantified using the simplified reference tissue (SRTM) model in the Pixel-wise Modeling Tool from PMOD, version 4.2. The cerebellum served as a reference region due to its limited dopamine-receptor expression. For subject-level analyses, parametric BP_ND_ images from both the sessions were co-registered to each participant’s MRI image as previously described [10,65].

Regions-of-interest (ROIs) were obtained in the ventral striatum (VS), caudate head, putamen, globus pallidus (GP), substantia nigra (SN), amygdala, caudo-medial orbitofrontal cortex (cmOFC), hypothalamus, insula, and anterior cingulate cortex (ACC). Bilateral subcortical ROIs of the VS, caudate head, putamen, SN, amygdala, and cerebellum were manually defined on the T_1_ MRI image according to established anatomical criteria. The hypothalamus was manually defined using a previously described method [66]. The cmOFC was manually defined using previously described definitions including Brodmann areas 14c and the posterior medial aspect of area 13 [10,67]. The GP was defined using segmentation provided by FSL (version 6.0, FMRIB Software Library).

### 4.5. Experimental Design

Each subject underwent a baseline general physical exam and assessment of PD severity utilizing the MDS-UPDRS parts II and III [61,62], assessment of impulsivity with a semi-structured interview, and completion of the QUIP-RS. We used a two-scan protocol to evaluate the effects of dAMPH on DA-R availability estimated with BP_ND_ and percent-change BP_ND_ relative to baseline. Following 72 h DAA medication withdrawal, the patients received placebo on the first experimental day and 0.43 mg/kg dAMPH on the day of the second scan, although the patients were informed that the order of placebo and drug would be randomized. The order of the scans was arranged to minimize any potential of dAMPH-induced changes to D2-R. Three hours after the single-blinded administration of treatment, the patients completed the PANAS, DEQ, and AIRS. PET and MRI images were also obtained as described above. All the patients were consistently monitored for possible adverse events throughout the experiment.

### 4.6. Statistics

Analyses were computed using R version 4.1.2 (R Foundation for Statistical Computing, Vienna, Austria). All the tests assumed non-normal distributions of data and considered the covariates of sex and a principal component (PC1) for age and UPDRS-III score, which assesses the severity of PD motor symptoms. The Wilcoxon signed-rank test assessed change in behavioral subscale scores in off- vs. on-dAMPH states; this assessment was performed for all the subjects, i.e., ICB+ subjects (PD-ICB) and ICB− subjects (PD-C). A mixed-effects model assessed the relationship between behavioral subscale scores and BP_ND_ within each ROI, with treatment (off- vs. on-dAMPH) as the repeated-measures variable. Spearman correlations assessed the relationship between QUIP-RS scores and percent change in behavioral subscale scores, and separately, the relationship between off-dAMPH BP_ND_ within each ROI and percent change in behavioral subscale scores. The results were controlled at a false discovery rate (FDR) of 0.05 to correct for multiple comparisons as used previously [58] and reported as p_CORR_ unless otherwise specified.

## 5. Conclusions

In conclusion, we found that, in PD, dAMPH exerts a net positive effect on mood that is mediated by DA neurotransmission in key mesocorticolimbic structures: ventral striatum (VS), caudate head, amygdala, and cmOFC. Impulsivity alters how PD patients perceive dAMPH mood effects and correlates with dAMPH-induced depression. Finally, baseline D2-R occupancy in the VS, caudate, and amygdala can predict dAMPH-induced improvements in mood. These results emphasize that, overall, the modification of the DA-ergic tone in the mesocorticolimbic circuit improves mood in PD, but these effects can also be influenced by pre-existing alterations in the reward neurocircuitry associated with ICBs. The role that these striatal and extra-striatal structures play in the overall affect warrants further investigation, especially given the prevalence of apathy and depression among PD patients [1,2]. Lastly, our study investigated the acute mood effects of DA release, although future studies are needed to explore how DAA therapies influence mood with chronic administration.

## Figures and Tables

**Table 1:** Results of linear mixed-effects model correlating questionnaire scores with regional BP_ND_.

	BP_ND_ (Regression Coefficient, *p*-Value Uncorrected, *p*-Value Corrected)			
Questionnaire	Caudate Head	Ventral Striatum	Amygdala	cmOFC
PANAS positive	−0.019, 0.32, 0.48	−0.055, 0.03 *, 0.28	−0.004, 0.33, 0.48	−0.027, 0.11, 0.28
DEQ feel	−0.009, 0.02 *, 0.12	−0.012, 0.03 *, 0.16	−0.001, 0.27, 0.41	−0.006, 0.07, 0.23
DEQ high	−0.008, 0.05 *, 0.47	−0.010, 0.11, 0.47	−0.001, 0.24, 0.47	−0.004, 0.28, 0.47
AIRS total	−0.005, 0.062, 0.16	**−0.011, 0.003, 0.03**	**−0.001, 0.024, 0.07**	**−0.006, 0.014, 0.07**

Only regions of interest with significant findings are shown in the table; significant comparisons (p**_CO_**_RR_ < 0.05) are shown in bold, comparisons that are significant before multiple comparisons are shown with an asterisk. cmOFC: caudo-medial orbitofrontal cortex.

**Table 2:** Demographic and clinical evaluation of PD participants.

Variables	All PD	ICB+ (PD-ICB)	ICB− (PD-C)	Test Statistic, *p* (PD-ICB vs. PD-C)
N	20	10	10	-
Sex (M/F)	12/8	7/3	5/5	0.833, 0.361
Age (yrs)	64.1 ± 5.78	65.8 ± 6.60	62.4 ± 4.53	2.12, 0.198
Disease duration (yrs)	6.43 ± 3.07	6.10 ± 2.28	6.75 ± 3.81	2.13, 0.650
MDS-UPDRS-III (off-dAMPH)	28.7 ± 13.1	27.6 ± 12.4	29.7 ± 14.3	2.10, 0.730
Total LEDD (mg/day)	671 ± 302	671 ± 314	672 ± 306	2.10, 0.994
QUIP-RS	26.0 ± 13.9	30.0 ± 12.0	19.9 ± 12.1	**1.88, 0.038**

Data are shown as mean ± standard deviation. Statistical tests: chi-squared test (sex); non-parametric *t*-test (age, disease duration, MDS-UPDRS-III, Total LEDD, QUIP-RS). Significant comparisons (*p*-values < 0.05) are shown in bold. Abbreviations: MDS-UPDRS: Movement Disorders Society-Unified Parkinson’s Disease Rating Scale; LEDD: levodopa equivalent daily dose; QUIP-RS: Questionnaire for Impulsive–Compulsive Disorders in Parkinson’s Disease Rating Scale.

## Data Availability

The raw data supporting the conclusions of this article will be made available by the authors on request.

## Abbreviations

The following abbreviations are used in this manuscript:

ACC	Anterior cingulate cortex
AIRS	Amphetamine Interview Rating Scale
BPND	Non-displaceable binding potential
cmOFC	Caudo-medial orbitofrontal cortex
DA	Dopamine
DAA	Dopamine agonist
dAMPH	Dextro-amphetamine
DAT	Dopamine transporter
DA-R	Dopamine receptor
DEQ	Drug Effects Questionnaire
FDR	False discovery rate
GP	Globus pallidus
MD-UPDRS	Movement Disorders Society—Unified Parkinson’s Disease Rating Scale
MRI	Magnetic resonance imaging
MoCA	Montreal Cognitive Assessment
QUIP-RS	Questionnaire for Impulsive–Compulsive Disorders in Parkinson’s Disease Rating Scale
PANAS	Positive and Negative Affect Scale
PC	Principal component
PD	Parkinson’s disease
PD-ICB	Parkinson’s disease with impulsive–compulsive behaviors
PD-C	Parkinson’s disease control
PET	Positron emission tomography
ROI	Region of interest
SRTM	Simplified reference tissue model
VS	Ventral striatum
